# Changes in gynecologic and breast cancer diagnoses during the first wave of the COVID-19 pandemic: analysis from a tertiary academic gyneco-oncological center in Germany

**DOI:** 10.1007/s00404-021-06211-7

**Published:** 2021-09-07

**Authors:** Till Kaltofen, Friederike Hagemann, Nadia Harbeck, Rachel Wuerstlein, Bernd Peter Kost, Alexander Burges, Sven Mahner, Fabian Trillsch

**Affiliations:** Department of Obstetrics and Gynecology, University Hospital and CCC Munich, LMU Munich, Marchioninistrasse 15, 81377 Munich, Germany

**Keywords:** Gynecologic cancer, Breast cancer, COVID-19, SARS-CoV-2

## Abstract

**Purpose:**

With the beginning of 2021, the world has been suffering from the COVID-19 pandemic for more than 1 year. More and more, we are able to evaluate side effects of the pandemic in the healthcare sector. A negative impact on cancer diagnoses is one of them. Careful observation of trends in an academic gyneco-oncological context appears important to identify potential negative developments.

**Methods:**

We analyzed the case number of gynecologic and breast cancer diagnoses in the period from January to June 2020 compared to 2019 and during the period of the first general German lockdown (March 22nd until May 5th 2020). Patients were characterized by age, tumor type, FIGO or TNM stage and presence of symptoms at initial hospital presentation.

**Results:**

The frequency of newly diagnosed gynecologic and breast cancer cases from beginning of January until end of June changed by − 10% and by − 12% during the lockdown in 2020 compared to 2019. In both periods, reduction of breast cancer cases was relatively larger than decrease of gynecologic cancers. Moreover, median patient age decreased. For the first half of 2020, we found a shift towards higher tumor stages (N+/M1 or FIGO III–IV). During the lockdown period, the appearance of tumor-associated symptoms at diagnosis increased by about 12%.

**Conclusion:**

This analysis illustrates the anticipated general decrease in diagnoses of primary cancers during the lockdown periods in 2020 due to COVID-19 pandemic for gynecologic and breast cancer cases.

## Introduction

In 2020, the world experienced the pandemic spread of a novel virus, known as severe acute respiratory syndrome coronavirus 2 (SARS-CoV-2) [1]. This virus pandemic claimed immense healthcare resources [2]. By February 1st, 2021, there were more than 100 million confirmed cases worldwide and over 2 million deaths due to the coronavirus disease 2019 (COVID-19) [3]. Consequently, besides departments that primarily treat COVID-19 all other areas of medicine were affected by social distancing restrictions, patients with a SARS-CoV-2 infection as a comorbidity and a massive shift of resources towards the fight against COVID-19.

Gyneco-oncologic patients, as well as patients with other malignancies, experience a stressful dichotomy during the ongoing pandemic. On the one hand, cancer patients are considered a high-risk group if infected by SARS-CoV-2 [4–8]. In one of the largest reports from Tian et al., they included data from 13,077 patients with COVID-19, including 232 patients who also had cancer. Compared to non-cancer patients, their risk for a severe course was 64% versus 32% and 20% versus 11% for death [8]. On the other hand, oncological patients have medical and psychological needs for therapy and are not able to completely avoid direct healthcare interactions. There is an active debate throughout oncologic societies and disciplines on how to deal with the medical repressions and limitations caused by the pandemic. Possible approaches could be a delay in surgery, a de-escalation of surgical radicality and a shift towards telemedicine [9, 10]. For breast cancer care, this would prompt an accentuation of neoadjuvant chemotherapy in early triple-negative and HER2+ carcinomas and preoperative endocrine therapy in luminal-like cancers [11, 12]. Gynecologic cancer treatment offers different adjustments depending on the histological entity—for example: endocrine therapy in early-stage low-risk endometrial cancer, hypofractionation of radiotherapy to reduce patient hospital appointments for cervical cancer, neoadjuvant chemotherapy in ovarian cancer and many more [13, 14].

Fortunately, in Germany, there was no need to substantially delay necessary oncological therapies according to relatively low numbers of COVID-19 patients compared to European hot spot areas as Italy, Spain, France or the UK during the first wave of the pandemic. Nevertheless, the quality of supportive, follow-up, palliative and loving care through medical staff decreased [15, 16]. Moreover, outpatient appointments declined as patients became worried and virtual assessments were promoted [9, 14]. Routine checkups, as the recommended yearly gynecologic examination from the age of 20, were frequently postponed—especially during the general lockdown in Germany lasting for 6.5 weeks from March 22nd until May 5th 2020. Almost in parallel, from March 26th until May 3rd no screening invitations for biennial mammography from the age of 50–69 years were sent [17].

Consequently, less cancer diagnoses in Germany in 2020 overall and particularly during the first lockdown period would be expected. A first study from a private German hospital group confirmed this in their facilities [18]. Studies from other European countries also support this hypothesis for cancer in general [19] as well as for gynecologic and breast cancer [20, 21]. So far, maybe attributable to a delay and a potential inertia in the recording system of large independent nationwide organizations, we are still waiting for sufficient representative data for Germany as a whole, answering this question. To improve the database with respect to the ongoing pandemic in cancer care departments, we analyzed the development of gynecologic and breast cancer diagnoses during the first half of 2020 compared to 2019 in our academic tertiary center for gynecologic and breast cancer at the LMU Munich University Hospital.

## Methods

In this quality assurance project, we included all patients diagnosed with gynecologic or breast cancer (including in situ stages) who presented to our academic center at LMU Munich (invasive cancer cases in 2019 in total: *n* = 509) between January and June 2020 compared to the same period in 2019. Our data are based on the specifications of the national certification commission of the German Cancer Society. We analyzed the development of cancer diagnoses during this half-year period and additionally focused on the period of the first general lockdown (March 22nd until May 5th 2020) in Germany compared to the same period in 2019. During these 6.5 weeks in 2020, every commercial service, not essential for the daily living, was closed. Only necessary errands, travel to work as well as taking a walk or sports outside within the own household group and a maximum of one additional person were allowed (social distancing). In our academic center, there was no reduction in consultation hours and surgery capacity for cancer cases during the entire period observed. Nursing and medical staff remained unchanged as well throughout the whole period.

For the descriptive analysis, patients were characterized by age, tumor type, Fédération Internationale de Gynécologie et d'Obstétrique (FIGO) or TNM stage and the presence of symptoms at initial hospital presentation. We considered abdominal swelling, pain, inflammation signs, a vaginal bleeding or a palpable mass of the breast as tumor-associated symptoms.

For descriptive and statistical analyses, we used SPSS Statistics 26 (IBM, Chicago, IL, USA). We compared the median age using the Mann–Whitney-*U* test. Other features were tested by Chi-square statistic. A *p* value beneath 0.05 was considered to be statistically significant. GraphPad Prism 8 (GraphPad Software, La Jolla, CA, USA) was used to prepare the figures.

## Results

**Fig. 1:**
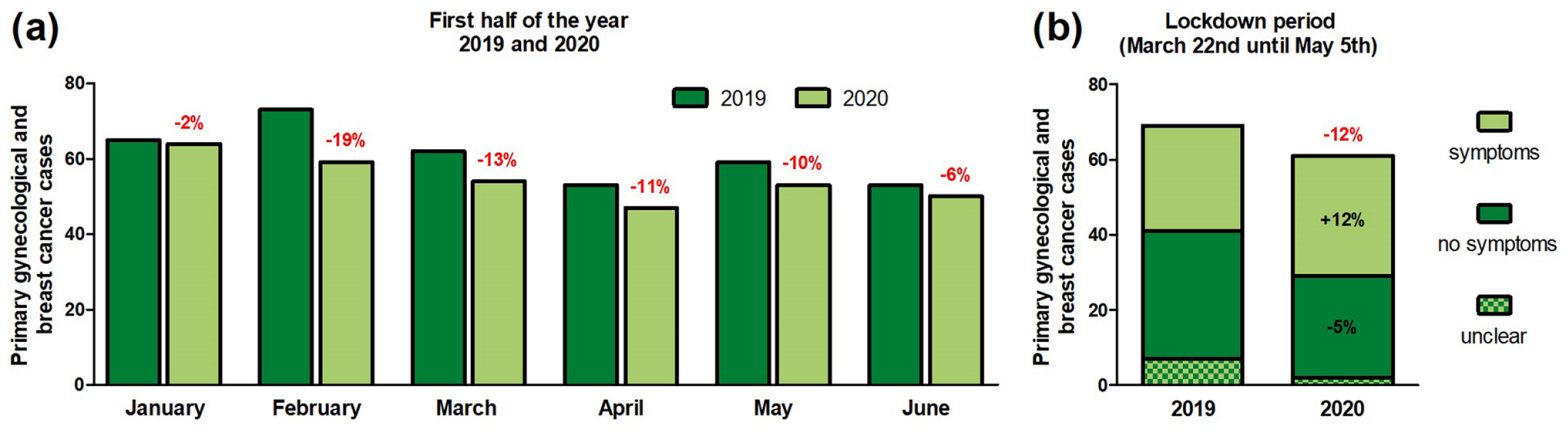
Decrease of primary gynecologic and breast cancer cases at LMU Munich: **a** from January until June 2020 compared to the same period in 2019. **b** During the first German lockdown (March 22nd until May 5th 2020), caused through the SARS-CoV-2 pandemic, compared to the same period in 2019 and the relative amount of patients with or without tumor-associated symptoms

We recorded 327 newly diagnosed gynecologic and breast cancer cases in our department from the beginning of January until the end of June in 2020 compared to 365 during the same period in 2019. This represents a decrease of − 10%. Gynecologic cancer diagnoses decreased by − 9% (*n* = 195 versus *n* = 177) and breast cancer diagnoses by − 12% (*n* = 170 versus *n* = 150) during the first half of 2020 compared to 2019 (Table 1). While we noticed only a slight decrease in January (− 2%) and June (− 6%), this was more pronounced in the months of February (− 19%), March (− 13%), April (− 11%) and May (− 10%) resulting a relevant decrease in cancer numbers at our gyneco-oncological department (Fig. 1a). The median age at first diagnosis decreased from 56.6 to 54.7 years, which was not statistically significant. Except for the subgroup of ovarian/ tubal/ peritoneal cancer and borderline tumors (+ 35%), diagnoses of all other cancer entities decreased (breast: − 12%, endometrial: − 37%, cervical: − 3%, vulvar/vaginal: − 45%, others: − 25%). According to the general change in the number of diagnoses, the differences for a single subgroup did not reach statistically significance. Most entities showed a tendency towards a decreased percentage of early TNM and FIGO stages (N0/M0 or FIGO I–II) favoring an increase of advanced diseases (N+/M1 or FIGO III–IV) (Table 1).

**Table 1:** Primary gynecologic and breast cancer cases with patient characteristics at LMU Munich from January until June 2020 compared to the same period in 2019

	First half of 2019	First half of 2020	Absolute difference (%)	Relative difference (%)
Cancer diagnoses	365	327	− 10	
Age (median)	56.6	54.7		
Breast cancer				
Total	170	150	− 12	
Tis	12 (7%)	11 (7%)		0
T1	53 (31%)	46 (31%)		− 1
T2–4	45 (27%)	30 (20%)		− 7
N+	41 (24%)	46 (31%)		7
M1	19 (11%)	17 (11%)		0
Ovarian/tubal/peritoneal cancer and borderline tumors				
Total	37	50	35	
FIGO I–II	13 (35%)	14 (28%)		− 7
FIGO III–IV	24 (65%)	36 (72%)		7
Endometrial cancer				
Total	19	12	− 37	
FIGO I–II	15 (79%)	9 (75%)		− 4
FIGO III–IV	4 (21%)	3 (25%)		4
Cervical cancer				
Total	87	84	− 3	
Tis	68 (78%)	69 (82%)		4
FIGO I–II	12 (14%)	8 (10%)		− 4
FIGO III–IV	7 (8%)	7 (8%)		0
Vulvar/vaginal cancer				
Total	40	22	− 45	
Tis	24 (60%)	13 (59%)		− 1
FIGO I–II	11 (28%)	6 (27%)		0
FIGO III–IV	5 (13%)	3 (14%)		1
Other cancers	12	9	− 25

Focusing on the lockdown period from March 22nd until May 5th 2020 compared to the same period 2019, the number of cancer diagnoses changed by − 12% (*n* = 69 versus *n* = 61) (Fig. 1b) as well as in both subgroups: gynecologic cancer − 5% (*n* = 39 versus *n* = 37) and breast cancer − 20% (*n* = 30 versus *n* = 24). In accordance to our results for the half-year period, median age of newly diagnosed cancer patients decreased during the lockdown from 57.8 to 50.8 years (Table 2). For this 6.5-week duration, tumor-associated symptoms at referral or admission to our hospital were noted for 12% more patients in 2020 compared to 2019. In contrast to the half-year analysis, no clear shift to higher tumor stages was evident when focusing on the lockdown period (Fig. 1b and Table 2). All changes of this analysis did not reach statistical significance.

**Table 2:** Primary gynecologic and breast cancer cases with patient characteristics at LMU Munich during the first German lockdown (March 22nd until May 5th 2020), caused through the SARS-CoV-2 pandemic, compared to the same period in 2019

	Control period 2019	Lockdown 2020	Absolute difference (%)	Relative difference (%)
Cancer diagnoses				
Total	69	61	− 12	
Breast	30	24	− 20	
Gynecologic	39	37	− 5	
Tis	19 (28%)	20 (33%)		5
M0 or FIGO I–III	41 (59%)	33 (54%)		− 5
M1 or FIGO IV	9 (13%)	8 (13%)		0
Age (median)	57.8	50.8		
Symptoms				
Yes	28 (41%)	32 (53%)		12
No	34 (49%)	27 (44%)		− 5
Unclear	7 (10%)	2 (3%)		− 7

## Discussion

In this analysis of a large German tertiary academic cancer center, a decrease in the number of primary cancer diagnoses was seen in our gyneco-oncological department during the first half of 2020 and also during the first German nationwide lockdown, compared to the same periods in 2019.

This decrease in numbers really started in February following the report of the first SARS-CoV-2-positive cases in Germany on January 27th [22] with almost 20% and partially returned back to the normal level by June 2020. As other studies suggest, these developments seemed most likely to be related to the COVID-19 pandemic [18–21]. Obviously, this is probably not a true reduction of cancer incidence during the lockdown as seen it in seasonal infectious diseases [23, 24] but rather a delay in detection or initial hospital presentation of primary cancers. The major decline of cases already in February and not only during the lockdown, might be explained through an upcoming great uncertainty in the population directly after the first German COVID-19 cases, which were detected in employees of a factory near Munich [22] but must be interpreted with caution in this single-center analysis. Nevertheless, our center did not have to reduce personnel or therapies offered during the period analyzed.

The observed decrease of cancer diagnoses might be caused by a multifactorial process. Routine checkups should be postponed and a pronunciation of telemedicine was demanded during the first pandemic wave according to authorities’ recommendations, studies from cancer departments [9, 10] and medical guidelines [16, 25]. Consequently, the amount of personal appointments decreased followed by a reduction of accuracy and power of secondary prevention. Interestingly, throughout the periods we analyzed, the drop of breast cancer cases was overrepresented compared to that observed in gynecologic cancer cases (lockdown: − 20% versus − 5%, first half-year: − 12% versus − 9%). This might have been caused by the suspension of the mammography screening invitations from March 26th until May 3rd in parallel to the German lockdown [17]. In contrast, for gynecologic cancers, there is no established screening program for all entities potentially leading to less relative reduction. In addition, the overall healthcare capacities for treatment of non-COVID-19 patients were reduced to promote enough infrastructure for SARS-CoV-2 positive patients. A prospective study in 18 German comprehensive cancer centers, analyzing the different dimensions of cancer treatment revealed restrictions up to 20% in diagnostic procedures with a peak in the second half of April 2020. The treatment itself whereas was nearly not affected. Major restrictions were seen in supportive, follow-up and palliative care [15, 16]. Besides official recommendations and structural changes, a third aspect is patient concerns about an elevated risk for SARS-CoV-2 infections in medical facilities. Eventually, it is triggered by a potentially higher susceptibility for cancer patients getting sick from COVID-19 [4]. Actual register studies show a significantly higher mortality for cancer patients with SARS-CoV-2 infections compared to non-cancer patients. However, the discussion about how to account for the usual co-morbidities and circumstances (for example higher age) in these analyses is still ongoing. Meanwhile, data from the European LEOSS register support a higher mortality rate of cancer patients compared to matched counterparts (23% versus 14%, *p* < 0.001) but show a lower mortality rate than most other studies [26]. Interestingly, a large US study cohort from New York with patients with gynecologic cancer and a SARS-CoV-2 infection did not detect any association between cytotoxic chemotherapy or cancer-directed surgery and COVID-19 severity or death. Only recent immunotherapy was associated with an increased risk. Their case fatality rate was only 14% [27].

Besides changes in the general number of cancer patients who presented to our department, their characteristics also changed. During both periods (half-year and lockdown), median patient age decreased, which could be expected [18, 21] as age is one of the main risk factors for severe COVID-19 [26, 28, 29] leading to a significantly reduced mobility of elderly people according to federal recommendations. Moreover, we interpreted a shift towards more advanced tumor stages, seen in the half-year analysis, as well as the 12% rise of patients with tumor-associated symptoms during the lockdown period as a sign for an increased perceived urgency of their disease, which forced them to visit a hospital and not being able to avoid contact to other potential infectious patients.

Aiming to avoid a delay in secondary prevention strategies, our center started multiple initiatives. We implemented an e-mail newsletter for gynecologic outpatient services, to inform on changes in our department as a consequence of the current pandemic situation. In parallel, our homepage was modified to provide information about this topic. Contact options were clearly explained and the modified concept in compliance with the required social distancing restrictions was comprehensively presented. One of them was a newly established consultation by phone and later by video conference, which raised interest and great acceptance.

The single-center setup can be seen as a potential limitation of our evaluation as it might not be representative for the whole gyneco-oncological sector in Germany. But it allows for a thorough detailed analysis that would not be possible in national and international surveys, hence it can also be seen as potential strength. Nationwide data from the German Cancer Society are to be expected in summer 2021.

## Conclusion

Our present analysis emphasizes the implications of the COVID-19 pandemic with a general decrease of primary cancer diagnoses, demonstrated here by the experiences of a large German tertiary academic gyneco-oncological center. Our findings should lead to a re-evaluation of our secondary prevention strategies and their logistics. Even if German healthcare is currently still capable to offer every necessary somatic cancer treatment during the ongoing pandemic, we may be in danger of missing the first important step when treating a malignant disease: an early diagnosis.

## Abbreviations

COVID-19: Coronavirus disease 2019
FIGO: Fédération Internationale de Gynécologie et d'Obstétrique
SARS-CoV-2: Severe acute respiratory syndrome coronavirus 2

## References

[1] World Health Organization (2020) Statement on the second meeting of the International Health Regulations (2005) Emergency Committee regarding the outbreak of novel coronavirus (2019-nCoV). https://www.who.int/news/item/30-01-2020-statement-on-the-second-meeting-of-the-international-health-regulations-(2005)-emergency-committee-regarding-the-outbreak-of-novel-coronavirus-(2019-ncov). Accessed 01 Feb 2021

[2] World Health Organization (2020) Coronavirus disease (COVID-19) pandemic. https://www.who.int/emergencies/diseases/novel-coronavirus-2019. Accessed 01 Feb 2021

[3] Johns Hopkins Coronavirus Resource Center (2020) COVID-19 Dashboard by the Center for Systems Science and Engineering (CSSE) at Johns Hopkins University (JHU). https://coronavirus.jhu.edu/map.html. Accessed 01 Feb 2021

[4] Dai M, Liu D, Liu M (2020). Patients with cancer appear more vulnerable to SARS-CoV-2: a multicenter study during the COVID-19 outbreak. Cancer Discov.

[5] Kuderer NM, Choueiri TK, Shah DP (2020). Clinical impact of COVID-19 on patients with cancer (CCC19): a cohort study. Lancet.

[6] Liang W, Guan W, Chen R (2020). Cancer patients in SARS-CoV-2 infection: a nationwide analysis in China. Lancet Oncol.

[7] Mehta V, Goel S, Kabarriti R (2020). Case fatality rate of cancer patients with COVID-19 in a New York hospital system. Cancer Discov.

[8] Tian J, Yuan X, Xiao J (2020). Clinical characteristics and risk factors associated with COVID-19 disease severity in patients with cancer in Wuhan, China: a multicentre, retrospective, cohort study. Lancet Oncol.

[9] El-Shakankery KH, Kefas J, Crusz SM (2020). Caring for our cancer patients in the wake of COVID-19. Br J Cancer.

[10] Kling SM, Philp MM (2020) The effects of the COVID-19 pandemic on oncological surgery. J Surg Case Rep 22:157. 10.1093/jscr/rjaa157 10.1093/jscr/rjaa157 PMC7243827 32467751

[11] Casella D, Fusario D, Cassetti D (2020). The patient’s pathway for breast cancer in the COVID-19 era: an Italian single-center experience. Breast J.

[12] Harbeck N (2021). Risk-adapted adjuvant therapy of luminal early breast cancer in 2020. Curr Opin Obstet Gynecol.

[13] Alkatout I, Karimi-Zarchi M, Allahqoli L (2020). Gynecological cancers and the global COVID-19 pandemic. J Turk Ger Gynecol Assoc.

[14] Ramirez PT, Chiva L, Eriksson AGZ (2020). COVID-19 global pandemic: options for management of gynecologic cancers. Int J Gynecol Cancer.

[15] Fröhling S, Arndt V (2020). Corona-Effekt in der Onkologie. Dtsch Arztebl.

[16] Society of Gynecologic Oncology (2020) COVID-19 resources for health care practitioners. https://www.sgo.org/practice-management/covid-19/. Accessed 01 Feb 2021

[17] Gemeinsamer Bundesausschuss (2020). Beschluss des Gemeinsamen Bundesausschusses über die befristete Aussetzung der Einladung zum Mammographie-Screening.

[18] Reichardt P, Bollmann A, Hohenstein S et al. (2020) Decreased Incidence of Oncology Admissions in 75 Helios Hospitals in Germany during the COVID-19 Pandemic. Oncol Res Treat. Doi: 10.1159/000512935 10.1159/000512935 PMC7801981 33333506

[19] Dinmohamed AG, Visser O, Verhoeven RHA (2020). Fewer cancer diagnoses during the COVID-19 epidemic in the Netherlands. Lancet Oncol.

[20] Gathani T, Clayton G, MacInnes E (2020). The COVID-19 pandemic and impact on breast cancer diagnoses: what happened in England in the first half of 2020. Br J Cancer.

[21] Tsibulak I, Reiser E, Bogner G (2020). Decrease in gynecological cancer diagnoses during the COVID-19 pandemic: an Austrian perspective. Int J Gynecol Cancer.

[22] Rothe C, Schunk M, Sothmann P (2020). Transmission of 2019-nCoV infection from an asymptomatic contact in Germany. N Engl J Med.

[23] aerzteblatt.de (2021) SARS-CoV-2: Weniger Fälle anderer Infektions­krankheiten gemeldet. https://www.aerzteblatt.de/nachrichten/120015/SARS-CoV-2-Weniger-Faelle-anderer-Infektionskrankheiten-gemeldet. Accessed 01 Feb 2021

[24] Iacobucci G (2020). Covid lockdown: England sees fewer cases of colds, flu, and bronchitis. BMJ.

[25] onkopedia (2020) Coronavirus-Infektion (COVID-19) bei Patienten mit Blut- und Krebserkrankungen. https://www.onkopedia.com/de/onkopedia/guidelines/coronavirus-infektion-covid-19-bei-patient-innen-mit-blut-und-krebserkrankungen/@@guideline/html/index.html. Accessed 01 Feb 2021

[26] ESCMID Emerging Infections Task Force (2020) Lean European Open Survey on SARS-CoV‑2 infected patients (LEOSS). https://leoss.net/. Accessed 01 Feb 2021 10.1007/s15010-020-01499-0 PMC7527665 33001409

[27] Lara OD, O'Cearbhaill RE, Smith MJ (2020). COVID-19 outcomes of patients with gynecologic cancer in New York City. Cancer.

[28] Imam Z, Odish F, Gill I (2020). Older age and comorbidity are independent mortality predictors in a large cohort of 1305 COVID-19 patients in Michigan, United States. J Intern Med.

[29] Richardson S, Hirsch JS, Narasimhan M (2020). Presenting characteristics, comorbidities, and outcomes among 5700 patients hospitalized with COVID-19 in the New York City area. JAMA.

